# Tellurite and Tellurate Reduction by the Aerobic Anoxygenic Phototroph *Erythromonas ursincola*, Strain KR99 Is Carried out by a Novel Membrane Associated Enzyme

**DOI:** 10.3390/microorganisms5020020

**Published:** 2017-04-19

**Authors:** Chris Maltman, Lynda J. Donald, Vladimir Yurkov

**Affiliations:** Department of Microbiology, University of Manitoba, Winnipeg, MB R3T 2N2, Canada; ummaltma@myumanitoba.ca (C.M.); lynda.donald@umanitoba.ca (L.J.D.)

**Keywords:** tellurite, tellurate, tellurium, reduction, aerobic anoxygenic phototroph

## Abstract

*Erythromonas ursincola*, strain KR99 isolated from a freshwater thermal spring of Kamchatka Island in Russia, resists and reduces very high levels of toxic tellurite under aerobic conditions. Reduction is carried out by a constitutively expressed membrane associated enzyme, which was purified and characterized. The tellurite reductase has a molecular weight of 117 kDa, and is comprised of two subunits (62 and 55 kDa) in a 1:1 ratio. Optimal activity occurs at pH 7.0 and 28 °C. Tellurite reduction has a V_max_ of 5.15 µmol/min/mg protein and a K_m_ of 3.36 mM. The enzyme can also reduce tellurate with a V_max_ and K_m_ of 1.08 µmol/min/mg protein and 1.44 mM, respectively. This is the first purified membrane associated Te oxyanion reductase.

## 1. Introduction

Oxyanions of tellurium (Te), especially tellurite (TeO_3_^2−^), are poisonous not only to bacteria, but also to humans. This compound is lethal to most microorganisms at concentrations as low as 1 µg/mL [1], with toxicity believed to be a consequence of its properties as a strong oxidant [2,3]. However, some microbes are able to resist very high levels of tellurite through its reduction to elemental Te. Even though the capacity to carry out this oxyanion transformation has been known for quite some time, there are very few purified examples of responsible enzymes. In some bacteria, non-specific reduction is accomplished by nitrate reductases [4,5]. Thiol:disulfide oxidoreductase of *Rhodobacter capsulatus* [6] and GutS of *E. coli* [7], among others [8,9,10,11], also play a role in tellurite resistance and/or reduction. However, these enzymes are associated with low level resistance and it is not their primary function. Only one tellurite specific reductase, found in *Bacillus* sp., STG-83 has been published [12]. The cytoplasmic enzyme is 197 kDa, comprised of three subunits (66, 43, and 20 kDa), functions optimally at 35 °C, pH 8.0, and possesses a maximal velocity (V_max_) of 5.2 µmol/min/mg protein with a K_m_ (concentration of substrate to achieve one-half V_max_) of 2.6 mM [12]. This bacterium does exhibit increased tellurite resistance (~220 µg/mL) and is believed to be capable of dissimilatory anaerobic reduction, suggesting the enzyme may be involved in respiration. 

Reduction of metal(loid) oxyanions can help dispose of excess electrons through the re-oxidation of nicotinamide adenine dinucleotide (NADH), flavine adenine dinucleotide (FADH_2_), or quinones, therefore maintaining optimal redox poise in vivo as seen in cells of *R. capsulatus* and *R. sphaeroides* [13,14]. Another strategy, possessed by *R. capsulatus,* is based on reduced uptake of the tellurite oxyanion. Acetate permease is responsible for TeO_3_^2−^ influx [15,16,17], therefore, competition between the oxyanion and acetate gives rise to increased resistance. A related approach has been identified in *E. coli*, where a mutated phosphate transport system also resulted in enhanced resistance [18]. Finally, certain microorganisms can neutralize Te oxyanions through production of volatile organic tellurides such as dimethyltelluride [19], but this yields negligible removal. With so little information about specific tellurite reducing enzymes, more work with highly resistant and strongly reducing bacteria would be helpful to expand our knowledge.

Microbial metalloid oxyanion reduction is of great importance for biogeochemical Te cycling in nature. In both anaerobic and aerobic environments, many of these transformations are a direct result of bacterial enzymatic activity [20,21,22]. Human behaviours have resulted in the release of vast amounts of toxic chemicals into the biosphere, including Te oxyanions, which contribute to serious pollution problems [23]. A potentially attractive and ecologically sound removal method is microbial bioremediation, which has been explored, but only to a very limited extent [24,25,26]. Therefore, the purification of specific enzymes able to reduce harmful Te oxyanions could aid in the development of environmentally friendly remediation strategies.

Aerobic anoxygenic phototrophs (AAP) are a group of bacteria which have an inherent high level resistance to tellurite [27]. Of all taxonomically described AAP, over half originate from extreme habitats, and of those tested, all are resistant to K_2_TeO_3_ [27]. Their minimum inhibitory concentrations (MICs) are significantly higher than in other studied tellurite reducers. The highest MICs for the purple non-sulfur bacteria *R. capsulatus* and *R. sphaeroides* are 800 and 900 µg/mL, respectively [13,14], whereas AAP possess MICs up to 2700 µg/mL [1]. Te crystals can be accumulated inside the cells of AAP [1], possibly indicating reduction by a cytoplasmic enzyme. However, many crystals are in close contact with the cell membrane, suggesting a likely membrane associated reductase. A recent publication confirmed that some AAP do possess constitutive tellurite reducing activity associated with membranes [28]. 

In this study, tellurite reduction in several AAP (*Erythromicrobium ezovicum*, strain E1; *Erythromicrobium ramosum*, E5; *Erythromonas ursincola*, KR99; and *Sandaracinobacter sibiricus*, RB 16–17) possessing high level resistance and the ability to reduce it to elemental tellurium (Te°) [1] was investigated. All species were originally isolated from the hot temperature springs in Russia [29,30,31,32,33].

## 2. Materials and Methods 

### 2.1. Tellurite Reductase Purification and Characterization

All species were grown under their optimal conditions, membranes collected, and TeO_3_^2−^ reductase activity confirmed by visual blackening, as described [28]. Membranes were homogenized in Tris HCl buffer, pH 7.8, treated with 2% Triton X-100 and incubated for 30 min at room temperature with gentle shaking, then filtered to remove debris using a 0.2 µm syringe filter. Solubilized membranes were tested for reductase activity by addition of 100 µg/mL tellurite and 25 µL of 10 mM NADH or 1 g/L of their respective carbon sources [28] to act as an electron donor(s). Only membrane fractions of strain KR99 retained activity and, therefore, it was used for all other experiments. The solubilized membranes were loaded onto a Superdex S-200 gel column previously equilibrated with 20 mM Tris HCl, pH 8.0, and eluted with a flow rate of 1.0 mL/min. The 2 mL fractions exhibiting tellurite reduction were pooled and concentrated with a 100 kDa membrane cut-off centrifugal concentrator and loaded onto a Source 15 Q anion exchange column equilibrated with 20 mM Tris HCl, pH 8.0. A 0–1 M NaCl gradient was used with a flow rate of 3.0 mL/min. The 2 mL fractions with reductase activity were pooled and concentrated as above. Native polyacrylamide gel electrophoresis (PAGE) (5%) confirmed the presence of only a single protein. Molecular weight was estimated with a Superdex S-200 gel filtration column equilibrated with 20 mM Tris HCl, pH 8.0. The protein standards used were aldolase (158 kDa), conalbumin (75 kDa), ovalbumin (43 kDa), carbonic anhydrase (29 kDa), ribonuclease A (13.7 kDa), and aprotinin (6.5 kDa). The column void volume was established with ferritin (440 kDa). Number and size of subunits was determined by SDS PAGE as published [34] with 5% stacking and a 12% running gels following denaturation by boiling for 4 min in 1% (*w*/*v*) sodium dodecyl sulfate (SDS) in the presence of 2-mercaptoethanol. Gels were stained with Coomassie brilliant blue [35]. 

### 2.2. Enzyme Properties and Kinetics

Glutamate, pyruvate, succinate, malate, lactate, acetate, and NADH were assayed for their capacity as electron donors for oxyanion reduction. Additionally, multiple metal(loid) oxyanions (tellurite, tellurate, selenite, selenate, meta- and orthovanadate) were tested as possible substrates. To study the tellurite reductase functional kinetics, the reaction mixture contained 500 µL Tris HCl pH 7.0, 25 µL NADH (5 mM), 50 µL purified enzyme (150 µg/mL), and tellurite (0–13 mM) or tellurate (0–5 mM). Activity was detected spectrophotometrically at 500 nm [36]. Optimal pH was determined in phosphate buffered saline (PBS) or Tris HCl buffer adjusted with 0.5 N NaOH or HCl to pH 6.0, 7.0, 8.0, or 9.0. The reaction mixture was incubated at various temperatures (20, 25, 28, 32, 35, 38, and 42 °C) to identify the optimum. Enzymatic activity (Velocity (V)) was defined in units (U), where 1 U is equal to 1 µmol substrate reduced per minute per mg protein (µmol/min/mg protein). Protein was measured by the Bradford method [37]. 

### 2.3. Mass Spectrometry

Protein samples from native gel slices were reduced, alkylated, and digested with trypsin as described [38]. Peptides eluted from the gel were mixed with an equal volume of DHB matrix (saturated 2,5-dihydroxybenzoic acid (DHB) in 50% ACN, 2% formic acid) on a metal target. Matrix assisted laser desorption/ionization (MALDI) spectra were obtained using a prototype quadrupole time-of-flight mass spectrometer built in the Physics and Astronomy department, University of Manitoba [39]. Spectral analysis was done with TOFMA and pTOOL, non-commercial software developed with the instruments. Ions with a signal/noise of 2 were selected, and the list was sent for identification by MASCOT [40]. All data were checked against the Swissprot and National Center for Biotechnology Information (NCBI) databases, limited to bacterial taxonomy and a 30 ppm error level. An open search using the same databases was also done for contaminants. 

## 3. Results

### 3.1. Physical Characteristics of Reductase

**Figure 1:**
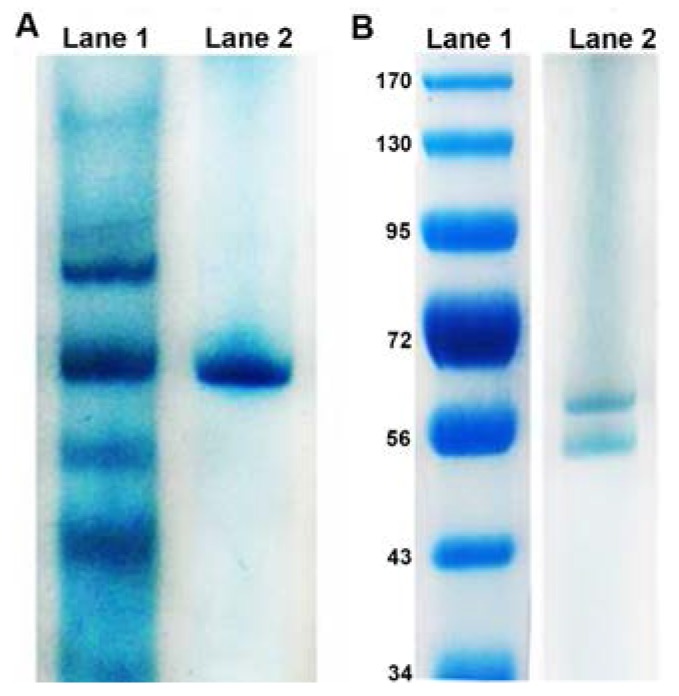
Protein purification from membranes of strain KR99. (**A**) Native PAGE: Lane 1, Solubilized membranes; Lane 2, Purified tellurite reductase. (**B**) SDS-PAGE: Lane 1, Molecular mass standards (in kDa); Lane 2, Tellurite reductase.

Upon collection and solubilisation of the membranes, strains E1, E5, and RB 16–17 could no longer reduce tellurite. It is likely that for these proteins, being released from the membrane resulted in a loss of proper conformation and therefore activity. However, KR99 samples retained the ability to reduce tellurite, with NADH as the optimal electron donor. Therefore, all further purification was performed using only this bacterium. Native gel analysis of the solubilized membranes revealed several protein bands prior to column chromatography (Figure 1A, Lane 1). Identification of the specific reducing enzyme was accomplished through size exclusion chromatography, followed by anion exchange chromatography, resulting in the 151-fold purification (Table 1) of a single protein, confirmed by native PAGE (Figure 1A, Lane 2). The molecular weight was 117 kDa and it is comprised of two subunits (62 and 55 kDa) in a 1:1 ratio (Figure 1B).

### 3.2. Biochemistry of Reductase 

**Figure 2:**
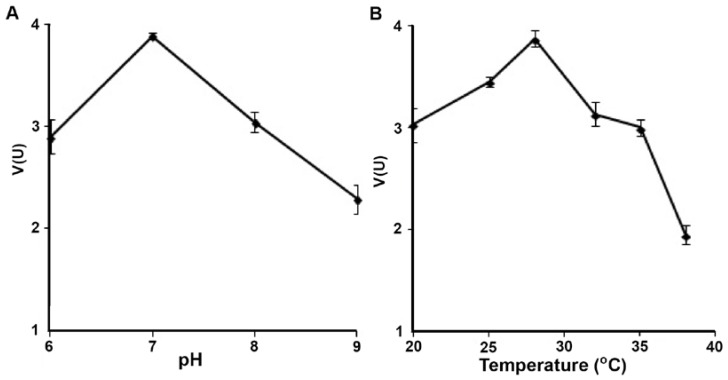
Effect of temperature (**A**) and pH (**B**) on tellurite reductase activity. Error bars represent one standard deviation.

**Figure 3:**
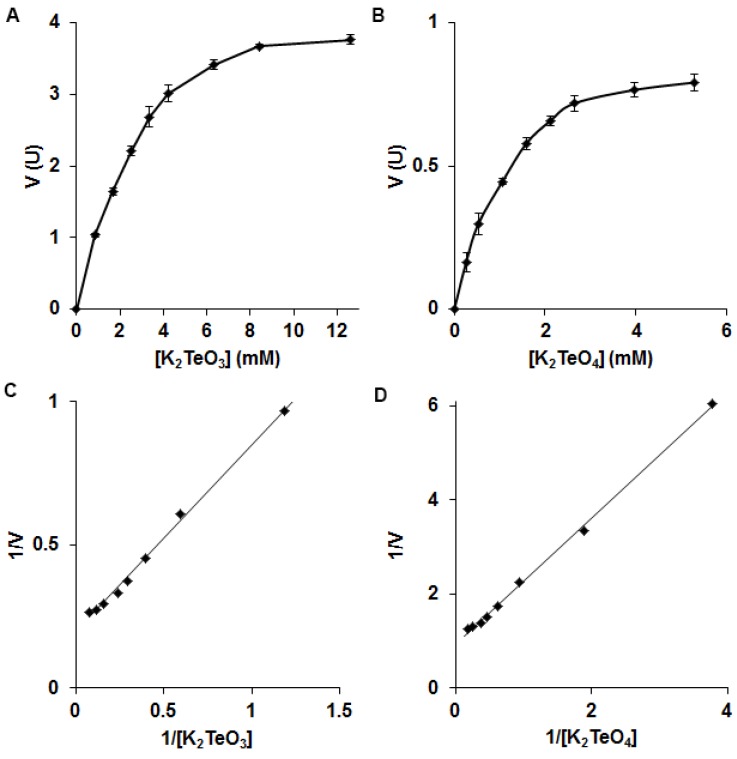
Michaelis–Menten plots for (**A**) Tellurite and (**B**) Tellurate. Lineweaver–Burk plots for (**C**) Tellurite and (**D**) Tellurate. Reactions were carried out at 28 °C, pH 7.0. Error bars represent one standard deviation.

The tellurite reductase exhibited its highest activity at 28 °C and pH 7.0 (Figure 2). Upon testing alternate metal(loid) oxyanions as substrates, the only other that could be reduced was tellurate. NADH, the only electron donor which could be used, had a K_m_ of 81.5 µM (Figure S1). From Lineweaver–Burk plots the K_m_ (obtained from slope of line) and V_max_ (obtained from y-intercept) values were calculated for each substrate. For tellurite reduction, values of 5.15 µmol/min/mg protein and 3.36 mM were estimated for V_max_ and K_m_, respectively (Figure 3A), whereas the V_max_ for tellurate was 1.08 µmol/min/mg protein with a K_m_ of 1.44 mM (Figure 3B).

### 3.3. Mass Spectrometry Analysis

Our first goal was to show we did isolate a protein, which was confirmed. According to mass spectrometry (MS) analysis of the tellurite reductase, it does not directly correspond to any currently known protein. The nearest match was the molecular chaperone GroEL from *Blastomonas* sp. CACIA14H2 (Figures S2 and S3) [41]. However, GroEL has yet to be characterized properly with its identity based solely on genomic DNA sequencing and homology to other similar proteins. Nothing else in the database fit with the required taxonomy, indicating that the reductase in this study is unique.

## 4. Discussion

In this work, we have isolated a membrane associated tellurite reductase from the AAP *E. ursincola*, strain KR99, which is also capable of reducing tellurate. The slower rate for tellurate reduction could be attributed to the need for more electrons to transform it to Te° as compared to conversion of tellurite. More time is required to accommodate the transfer of the extra electrons, hence, there is a decreased rate. This is the first example of its kind, as well as the first from an AAP. Unlike the one isolated from *Bacillus* STG-83, this enzyme is not used for anaerobic respiration as KR99 does not grow anaerobically. Attempting to draw comparisons between the tellurite/tellurate reductase from strain KR99 and other known Te oxyanion reducing enzymes is challenging as this protein is unique. It was isolated from a Gram-negative bacterium, membrane associated, and specific to Te oxyanions. The previously published tellurite reductase from *Bacillus* STG-83 appears to have a similar V_max_ and K_m_, however, it is much larger (197 kDa), comprised of three subunits, cytoplasmic in origin, and purified from a Gram-positive organism [12]. In some cases, nitrate reductases are implicated in tellurite reduction, therefore it could be argued the protein isolated here belongs to this family. However, KR99 is not capable of nitrate reduction, ruling out this option [32]. Mass spectrometry analysis confirmed the uniqueness of this reductase, as the closest match in the database was GroEL, but many ions were unidentified. In other bacteria, newly made enzymes are folded by GroEL [42], but in some cases, it has a secondary function [43], indicating that it can have multiple roles. This is not the whole answer, as what we know about GroEL in other organisms does not match the bands on the SDS gel from our protein. However, when we have the genome sequence of KR99, we can use the mass spectrometry fingerprint to search for the gene(s), make clones, and purify proteins at a larger scale. Then, proper characterization can be done as identification based on homology to other similar proteins is not the best approach. Its exact role in cells remains unclear, though strain KR99 grown in the presence of tellurite demonstrates an increase of biomass [28], attributing to some important physiological function. The fact that this reductase is NADH dependent could indicate that reduction of Te oxyanions is involved in disposal of electron excess through the re-oxidation of NADH, maintaining optimal redox poise in cells [13,14]. This permits optimal growth and explains the observed increase in biomass. As AAP are known to have an inherent ability to resist and reduce extremely high levels of different toxic metal(loid) oxyanions, especially tellurite [27], continued research may lead to ecologically friendly methods of bioremediation of these highly toxic substances.

## Figures and Tables

**Table 1:** Isolation of tellurite reductase from the membranes of *E. ursincola*, strain KR99.

Fraction	Activity (µM K_2_TeO_3_/min)	Total Protein (mg/L)	Specific Activity (µM K_2_TeO_3_/min/mg Protein)	Yield (%)	Fold Purification
Cell Lysate	8.14	261	0.031	100	1
Membranes	2.25	31	0.073	27.64	2.36
S200 fraction	1.87	0.64	2.94	22.97	94.84
Ion Exchange fraction	1.31	0.28	4.71	16.09	151.94

## Supplementary Materials

The following are available online at http://www.mdpi.com/2076-2607/5/2/20/s1, Figure S1: Michaelis–Menten (A) and Lineweaver–Burk (B) plots of the electron donor NADH for tellurite reduction. The reaction was carried out at 28 °C, pH 7.0. Error bars represent one standard deviation. Figure S2: MS sequence analysis of the membrane associated tellurite/tellurate reductase from strain KR99 compared to its nearest match GroEL from *Blastomonas* sp. CACIA14H2. Sequence identified by data matching is underlined., Table S1: Comparison of ions from the tryptic digest of KR99 tellurite reductase to those expected from a similar digest of the GroEL protein from *Blastomonas* sp. CACIA14H2 (NCBI gi|563284320).
