# Supplementary material for: Tellurite and Tellurate Reduction by the Aerobic Anoxygenic Phototroph Erythromonas ursincola, Strain KR99 Is Carried out by a Novel Membrane Associated Enzyme
Source: Microorganisms. 2017 Apr 19;5(2):20. doi: 10.3390/microorganisms5020020 (PMC5488091; doi:10.3390/microorganisms5020020)
Supplement: Supplementary file 1 [file microorganisms-05-00020-s001.pdf]

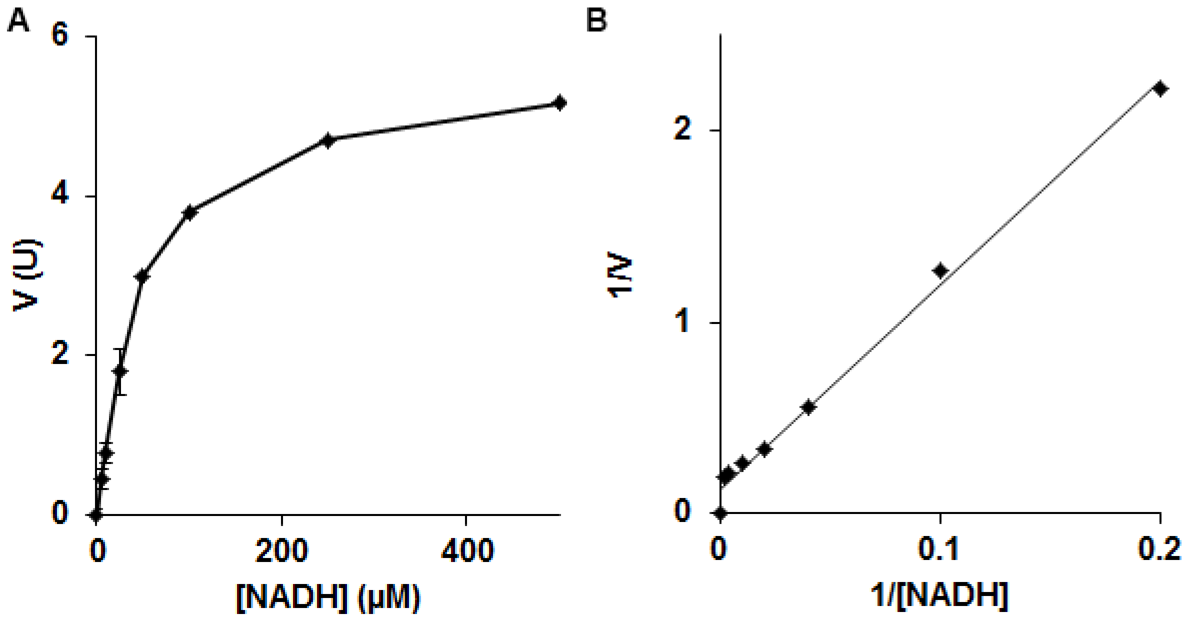


**Figure S1.** Michaelis–Menten (A) and Lineweaver–Burk (B) plots of the electron donor NADH for tellurite reduction. The reaction was carried out at 28 °C, pH 7.0. Error bars represent one standard deviation.

>gi|563284320|gb|ESZ88862.1| molecular chaperone GroEL [*Blastomonas* sp. CACIA14H2]

MAAKDVKFGRDARERILR<u>GVDILADAVKVTLGPKGRNVVIDKSFGAPR</u>ITKDGV
SVAKEIELKDKFENMGAQMVKEVASK<u>TNDIAGDGTTTATVLAQAIVR</u>EGMKSV
AAGMNPMDLKR<u>GIDLAVTKVVENLK</u>SRSK<u>DVAGSNEIAQVGIISANGDR</u>EVGEK
IAEAMERVGKEGVITVEEAK<u>GLEFELDVVEGMQFDRGYLSPYFITNPDKMTVEL</u>
<u>DNPYILIHEK</u>KLSNLQAMLPILEAVVQTGRPLLIIAEDIEGEALATLVVNKLRGGL
KVAAVKAPGFGDRRKAMLEDIAILSKGEMISEDLGIKLENVTLGMLGQAKR<u>VSI</u>
<u>DKDNTTIVDGAGEADAIK</u>ARVEAIRTQIDNTTSDYDREKLQERLAKLAGGVAVI
KVGGASEVEVKEKKDRVDDALHATR<u>AAVEEGIVPGGGTALLYATSALEGLTGE</u>
<u>NDDQTR</u>GIDIIRK<u>ALFAPVRQIAQNAGHDGAVVSGKLLDGNDPTLGFNAATDTY</u>
<u>ENLVAAGVIDPTKVVRAALQDAASVAGLLITTEAAICDAPEDK</u>AAAGGMGGMP
GGMGGMGGMDF

**Figure S2.** MS sequence analysis of the membrane associated tellurite/tellurate reductase from strain KR99 compared to its nearest match GroEL from *Blastomonas* sp. CACIA14H2. Sequence identified by data matching is underlined.

**Table S1**. Comparison of ions from the tryptic digest of KR99 tellurite reductase to those expected from a similar digest of the GroEL protein from *Blastomonas* sp. CACIA14H2 (NCBI gi|563284320).

| **Observed ions** | **Expected ions** | **Peptide** | | **MS/MS**[1] | **Sequence of *Blastomonas* GroEL** |
|---|---|---|---|---|---|
| 1302.687 | 1302.717 | 37 | 48 | okay | NVVIDKSFGAPR |
| 1498.822 | 1498.884 | 119 | 132 | | GIDLAVTKVVENLK |
| 1514.722 | 1514.753 | 198 | 210 | poor | GYLSPYFITNPDK |
| 1595.906 | 1595.937 | 19 | 34 | poor | GVDILADAVKVTLGPK |
| 1741.917 | 1742.018 | 119 | 134 | | GIDLAVTKVVENLKSR |
| 1814.905 | 1814.936 | 211 | 225 | | MTVELDNPYILIHEK |
| 1883.854 | 1883.885 | 182 | 197 | | GLEFELDVVEGMQFDR |
| 1978.157 | 1978.206 | 16 | 34 | no match | ILRGVDILADAVKVTLGPK |
| 1985.871 | 1985.989 | 137 | 156 | | DVAGSNEIAQVGIISANGDR |
| 2088.049 | 2088.094 | 81 | 101 | poor | TNDIAGDGTTTATVLAQAIVR |
| 2132.028 | 2132.072 | 323 | 343 | | VSIDKDNTTIVDGAGEADAIK |
| 2306.167 | 2306.237 | 446 | 468 | poor | ALFAPVRQIAQNAGHDGAVVSGK |
| 2714.309 | 2714.356 | 503 | 529 | | AALQDAASVAGLLITTEAAICDAPEDK |
| 2743.333 | 2743.386 | 135 | 161 | | SKDVAGSNEIAQVGIISANGDREVGEK |
| 3311.612 | 3310.671 | 198 | 225 | | GYLSPYFITNPDKMTVELDNPYILIHEK |
| 3375.608 | 3375.656 | 405 | 438 | excellent | AAVEEGIVPGGGTALLYATSALEGLTGENDDQTR |
| 3559.763 | 3559.828 | 469 | 502 | good | LLDGNDPTLGFNAATDTYENLVAAGVIDPTKVVR |
| 4172.104 | 4171.152 | 405 | 445 | | AAVEEGIVPGGGTALLYATSALEGLTGENDDQTRGIDIIRK |

[1]Tandem mass spectrometry to test whether the sequence of the peptides matched the expected.
